# In Vitro Differentiation of Human Amniotic Epithelial Cells into Hepatocyte-like Cells

**DOI:** 10.3390/cells11142138

**Published:** 2022-07-07

**Authors:** Marcin Michalik, Patrycja Wieczorek, Piotr Czekaj

**Affiliations:** Department of Cytophysiology, Chair of Histology and Embryology, Faculty of Medical Sciences in Katowice, Medical University of Silesia in Katowice, 40-752 Katowice, Poland; mmichalik@sum.edu.pl (M.M.); pszmytkowska@sum.edu.pl (P.W.)

**Keywords:** amniotic epithelial cells, stem cells, differentiation, hepatocytes, EGF, HGF

## Abstract

Human amniotic epithelial cells (hAECs) represent an interesting clinical alternative to human embryonic (hESCs) and induced pluripotent (hiPSCs) stem cells in regenerative medicine. The potential of hAECs can be enhanced ex vivo by their partial pre-differentiation. The aim of this study was to evaluate the effectiveness of 18-day differentiation of hAECs into endodermal cells, hepatic precursor cells, and cells showing functional features of hepatocytes using culture media supplemented with high (100 ng/mL) concentrations of EGF or HGF. The cells obtained after differentiation showed changes in morphology and increased expression of *AFP*, *ALB*, *CYP3A4*, *CYP3A7*, and *GSTP1* genes. HGF was more effective than EGF in increasing the expression of liver-specific genes in hAECs. However, EGF stimulated the differentiation process more efficiently and yielded more hepatocyte-like cells capable of synthesizing α-fetoprotein during differentiation. Additionally, after 18 days, GST transferases, albumin, and CYP P450s, which proved their partial functionality, were expressed. In summary, HGF and EGF at a dose of 100 ng/mL can be successfully used to obtain hepatocyte-like cells between days 7 and 18 of hAEC differentiation. However, the effectiveness of this process is lower compared with hiPSC differentiation; therefore, optimization of the composition of the medium requires further research.

## 1. Introduction

Liver failure, whether chronic or acute, often requires treatment by transplantation of this organ [1,2]. Unfortunately, global statistics show that some people waiting for the organ will not receive a transplant in time due to disease progression and a long waiting list [3,4]. In addition, the shortage of liver donors, insufficient for meeting the demand for transplants, necessitates the search for alternative therapies in regenerative medicine. One of its key directions is the use of native stem cells [5], and potentially also ex vivo modified stem cells, as an alternative or complementary therapy to organ transplantation.

Among pluripotent cells, the most popular are human embryonic stem cells (hESCs) derived from embryonic tissues [6] and induced pluripotent stem cells (hiPSCs) obtained from somatic cells by inducing the expression of specific transcription factors [7]. Despite many indisputable advantages, hESCs also have disadvantages such as harvesting difficulties related to their low abundance, isolation efficiency, and ethical controversies surrounding foetal sourcing and potential tumorigenicity when used in vivo [8]. It should be emphasized that hiPSCs have disadvantages as well, such as the tendency to accumulate chromosomal abnormalities, potential genetic instability, and the high costs and low efficiency of their collection [9,10].

So far, generally effective methods have been described to differentiate hESCs [11,12] and hiPSCs [13] into hepatoblasts and mature hepatocytes. It has been shown, among others, that the use of culture dishes coated with an extracellular matrix, especially Matrigel due to its properties, promotes the process of hESC and hiPSC differentiation into endodermal cells [14] and then into hepatocyte-like cells, most of which have the ability to synthesise albumin [15,16], which is an indicator of complete differentiation [12].

An alternative to these pluripotent cells could be human amniotic cells (hACs), which, in the amniotic membrane, form two main populations of cells exhibiting stem cell characteristics. More than 100 million human amniotic epithelial cells (hAECs) and a smaller number of human amniotic mesenchymal stromal cells (hAMSCs) can be isolated from the amnion of a single human placenta. Obtaining the amnion of the human placenta to isolate cells raises no moral or ethical controversies.

The population of epithelial cells is isolated by trypsinization of the amniotic membrane [17,18]. Some hAECs possess pluripotency surface markers, such as SSEA-3, SSEA-4, TRA-1-60, and TRA-1-81, and are characterised by the expression of pluripotent cell-specific genes: *POU5F1* (*OCT-4*), *NANOG*, *SOX-2*, *Lefty-A*, *FGF-4*, *REX-1*, and *TDGF-1*. hAECs originate from the epiblast, which gives rise to three germ layers. As a result, isolated hAECs can potentially give rise to tissues derived from all germ layers. Compared with hESCs or hiPSCs, hAECs are characterised by a low expression of major histocompatibility complex-I (MHC-I) agents and absence of major histocompatibility complex-II (MHC-II) agents, which limits the possibility of inducing an immune response in the recipient. In contrast, amniotic epithelial cells show high expression of the CD47 antigen responsible for the “do not eat me” signal and of the antigen complex for the CD55/CD59 complement regulatory proteins. This immunological privilege makes afterbirth cells good candidates for use in regenerative and transplant medicine, including the treatment of liver failure. Another unquestionable advantage of hAECs over other stem cell populations is their low telomerase activity, which is why they do not show tumorigenicity after in vivo [19].

Native hAECs have already been used in several animal models of experimental liver failure therapy [20,21,22,23]. Some studies have also used a medium collected from a hAEC culture containing their secretion products [24] and epithelial cells partially differentiated into human hepatocytes [25]. The use of amniotic cells and their derivatives in animal models of liver failure therapy resulted in improved organ function [26].

Previous reports [27] describing the attempts to differentiate hAEC into hepatocyte-like cells were based on a multistage differentiation under the conditions of supplementation with a mixture of several growth factors, such as epidermal growth factor (EGF), basic fibroblast growth factor 2 (bFGF2), basic fibroblast growth factor 4 (FGF4), and hepatocyte growth factor (HGF). The concentrations of growth factors used did not exceed 20 ng/mL [25,28,29,30]. hAECs differentiated in vitro into hepatocytes in a medium enriched with three growth factors: HGF, EGF, and FGF-2. However, without dimethyl sulfoxide (DMSO), they showed higher expression of genes characteristic of the foetal hepatoblast and hepatocyte lineages—*ALB*, *A1AT*, *CYP3A4*, *CYP3A7*, *CYP1A2*, *CYP2B6*, and *ASGPR1*—as compared with native cells [31,32].

Furthermore, some similarities between hAECs and iPSCs indicated that the iPSC differentiation protocol would be effective in differentiating hAECs. Studies on iPSCs [16] showed that a high concentration of 100 ng/mL HGF is highly effective in promoting the differentiation of pluripotent cells into hepatocytes, and the efficiency of this process, assessed as the percentage of cells capable of albumin synthesis, reaches 80%. On the other hand, it was suggested [29] that HGF at a concentration of 100 ng/mL itself would not be effective, but EGF at this concentration would be more effective in promoting the perinuclear localization of the HNF4a transcription factor, which is a key factor for cell differentiation towards endodermal lineage. Based on the abovementioned observations, we decided to compare both factors at high concentrations—higher than in previous attempts to differentiate hAEC.

A handful of scientific reports, e.g., in murine experimental models, have clearly shown that partial cell differentiation prior to in vivo application produces a better therapeutic effect in vivo than the use of native hAECs in the treatment of liver failure. hAECs partially differentiated into hepatocytes and administered to mice with chronic liver damage contributed to a higher increase in serum albumin level and an overall better improvement in liver function compared with the mice that received native hAECs [33]. It has been observed that it is possible to increase the effectiveness of hESC differentiation by using a sequential culture consisting of multistage differentiation. In the first stage, the action of factors such as wnt-3a and activin-A promotes cell differentiation into the endoderm lineage [11,34]; in the second stage, the action of growth factors promotes the differentiation of cells showing endodermal characteristics into hepatoblasts; in the third stage, the final maturation of hepatoblasts into hepatocytes occurs in the presence of steroids (sodium salts of hydrocortisone succinate, or dexamethasone, whose action promoting hepatoblast maturation to hepatocytes seems to be more effective) [16].

The aim of the presented research was to evaluate the effectiveness of the differentiation of epithelial cells isolated from the amnion of the human placenta into endodermal cells, hepatic precursor cells, and ultimately cells displaying characteristics of functional hepatocytes and/or cholangiocytes under conditions of supplementation with high concentrations of EGF or HGF. We used a modified protocol of hiPSC differentiation, whose initial potential for proliferation and differentiation into other cells is significantly higher as compared with hAECs.

## 2. Materials and Methods

### 2.1. Human Placenta

The placentas were collected from women aged 18–35 years after informed consent from the patient to collect the material for research. The placentas came from normal full-term pregnancies terminated by caesarean section for obstetric reasons (transverse position of the foetus, longitudinal pelvic position, foetopelvic disproportion) or for non-obstetric indications in the absence of signs and symptoms of placental insufficiency.

The inclusion criteria for the study were good health of the mother and child, uncomplicated pregnancy, and on-term delivery.

The exclusion criteria were smoking, drinking alcohol during pregnancy, inflammation, chronic non-obstetric conditions prior to or during pregnancy, and congenital malformations of the foetus.

Human placentas were collected with the consent of the Bioethical Committee of the Medical University of Silesia in Katowice (decision no. KNW/0022/KB/178-2/17).

### 2.2. Isolation of Amniotic Epithelial Cells

The amniotic membrane was manually separated from the chorion and then cut into pieces of approx. 1.5 × 1.5 cm. The pieces were enzymatically digested three times with trypsin (0.05%) supplemented with EDTA (Gibco, Waltham, MA, USA) for 30 min at a temperature of 37 °C. The cell suspension obtained after each digestion was centrifuged at 300× *g* for 5 min at 4 °C. The hAECs obtained from subsequent digestion stages were pooled, filtered through a 100 µm cell strainer, counted in an automatic cell counter (MOXI Z Mini Automated Cell Counter; Orflo, Ketchum, ID, USA), placed in a banking medium containing alpha-minimum essential medium (α-MEM), foetal bovine serum (FBS), and DMSO, then frozen in liquid nitrogen.

### 2.3. Cell Culture

After unbanking, hAECs were seeded at a density of 2.1 × 10^5^ cells/cm^2^ into culture dishes previously coated with the growth-factor-reduced Matrigel (Corning, New York, NY, USA) at a concentration of 0.125 mg/mL. The cultures were carried out in an incubator MCO-19M (Sanyo, Moriguchi, Japan) at 37 °C (5% CO_2_, 95% air, 95% relative humidity).

The control cultures were grown for 18 days in DMEM/F12 (PAN Biotech, Aidenbach, Germany) medium supplemented with 10% KnockOut Serum Replacement (KOSR) (Gibco), 1% solution of non-essential amino acids (NEAA) 100× (Gibco), 1% solution of L-glutamine (L-Glut) (Sigma-Aldrich, Darmstadt, Germany), 1% solution of antibiotic and antimycotic pen/strep 10,000 U/mL (Gibco), and 20 ng/mL of EGF (Peprotech, Cranbury, NJ, USA). The medium was changed every other day.

#### 2.3.1. hAEC Differentiation

Epithelial cells were differentiated using a modified protocol proposed by Carpentier et al. [16] for the purpose of hiPSC differentiation into hepatocytes. The original protocol provided for pre-differentiation of stem cells into definitive endoderm cells using the STEMdiff Definitive Endoderm Kit (STEMCELL Technologies, Vancouver, BC, Canada). The applied modification consisted of a two-stage differentiation by omitting the stage of pre-differentiation of hAECs into definitive endoderm cells and preforming direct differentiation into hepatoblasts (Table 1). For this purpose, after unbanking, hAECs were seeded in DMEM/F12 control medium supplemented with 10% KOSR, 1% NEAA, 1% L-Glut, 1% pen/strep, and 20 ng/mL EGF for 72 h of pre-culture. After this time, the cells were passaged and placed in DMEM/F12 differentiation medium no. 1, supplemented with 10% KSOR, 1% NEAA, 1% L-Glut, 1% pen/strep, 1% DMSO (Sigma-Aldrich), and 100 ng/mL EGF or 100 ng/mL HGF (Peprotech), depending on the test group. On day 15 of culture, after the assumed completed stage of hAEC differentiation into hepatoblasts, the second stage of culture began, which aimed to stimulate the maturation of hepatoblasts into hepatocytes. The cells were re-passaged and placed in DMEM/F12 differentiation medium no. 2, supplemented with 10% KOSR, 1% NEAA, 1% L-Glut, 1% pen/strep, and 10^−7^ M dexamethasone (Sigma-Aldrich). The medium was changed every other day, and the culture was completed on day 18.

#### 2.3.2. Collection of Cells for Research and Passage

During cultivation, cells were collected for analysis at four time points (after 72 h and on days 7, 15, and 18). Gentle Cell Dissociation Reagent (STEMCELL Technologies) was used as recommended by the manufacturer to dissociate adherent cells from the bottom of the culture dish, and DMEM/F12 medium was used to deactivate it. During cultivation, when the cells reached 90% confluence, they were passaged and seeded into a stage- and group-appropriate medium, supplemented with 10 µm of ROCK apoptosis inhibitor (STEMCELL Technologies).

### 2.4. Cytometric Analysis of Surface Markers

Adherent (after dissociation) and non-adherent hAECs were centrifuged together and suspended at a density of 1 million cells per 1 mL PBS supplemented with 10% FBS and 5 mM EDTA. Cell suspension in the amount of 100 µL (1 × 10^6^ cells/mL) was incubated for 30 min at 4 °C with specific antibodies: CD44-FITC (IM1219U), CD73-PE (B68176), CD90-AF750 (B36121), CD105-PC7 (B43293) (Beckman Coulter, Brea, CA, USA), SSEA-4-PerCP-Cy5.5 (561565) (BD Pharmingen, San Diego, CA, USA). Alternatively, appropriate fluorochrome-conjugated isotype controls were used: mouse IgG1-FITC (A07795), mouse IgG1-PE (A07796), mouse IgG1-APC-Alexa Fluor 750, mouse IgG1-PC7 (Beckman Coulter), and mouse IgG3 κ isotype-PerCP-Cy5.5 (BD Pharmingen). After incubation with antibodies, the cells were suspended in 300–400 µL PBS supplemented with 10% FBS and 5 mM EDTA and subjected to cytometric analysis using a Cytoflex flow cytometer (Beckman Coulter).

### 2.5. Cell Count and Viability Assessment

The cell number and viability were assessed using the ApoFlowEx FITC Kit (Exbio, Vestec, Czech Republic) each time before seeding the cells into culture dishes and at each of the four time points. For this purpose, pooled adherent and non-adherent hAECs were counted using a Cytoflex cytometer, and then batches of 200,000–500,000 cells were suspended in 100 µL of pre-diluted buffer (Annexin V Binding Buffer 10×). The cell suspension was supplemented with 5 µL Annexin V-FITC and 5 µL propidium iodine and incubated for 15 min at room temperature, followed by analysis on a Cytoflex cytometer.

### 2.6. Immunofluorescent Identification of Selected Proteins

After removal of the medium from the culture chambers, the cells were fixated by 15 min incubation with 4% paraformaldehyde (PFA) and subjected to permeabilisation of the cytoplasmic membrane by incubation in 0.25% Triton X-100 solution for 10 min at room temperature. Blocking of nonspecific protein binding sites was performed by incubating cells for one hour at room temperature in 0.1% PBS–Tween solution supplemented with 1% bovine serum albumin (BSA), 10% normal goat serum (NGS), and 0.3 M glycine. The cells were then incubated overnight at 4 °C with the following primary antibodies: Cytokeratin 7 Antibody (MA5-11986) (Invitrogen, Waltham, MA, USA), Anti-alpha 1 Fetoprotein Antibody (AFP-01) (Abcam, Cambridge, UK), Anti-Albumin Antibody (EPR20195) (Abcam), Anti-CYP7A1 Antibody (E-10): sc-518007 (Santa-Cruz, Dallas, TX, USA), Anti-CYP3A4 Recombinant Rabbit Monoclonal Antibody (JA11-33) (Invitrogen), Anti-Glutathione S-transferase-Pi monoclonal Antibody (USal-hGST-Pi-McAb-1) (Enzo, Farmingdale, NY, USA), and Anti-GSTA1 Antibody (orb157401) (Biorbyt, Cambridge, UK). In the next stage, the cells were incubated for one hour with two secondary antibodies: Goat anti-Mouse IgG (H + L), Alexa Fluor Plus 488 (Invitrogen), and Goat Anti-Rabbit IgG H&L Alexa Fluor^®^ 568 (Invitrogen) (at 4 °C). DAPI dye was used to stain the cell nuclei. To exclude nonspecific antibody binding, some cells were incubated with the appropriate isotype control.

The slides were evaluated using an Olympus BX43F fluorescence microscope (Olympus, Shinjuku, Japan). The percentage of positive cells was estimated at 100× magnification and scored on a five-point scale: (-)—no protein or single-positive cells < 1%; (+)—protein present in 0–25% cells; (++)—protein present in 26–50% cells; (+++)—protein present in 51–75% cells; and (++++)—protein present in 76–100% cells.

### 2.7. Assessment of the Ability of Cells to Secrete Albumin

At each time point, a portion of the medium was collected from control and test culture dishes. Freshly prepared media that were not used for cultivation served as negative controls. Human albumin was detected in culture media by the enzyme-linked immunosorbent assay (ELISA) using the Albumin Human ELISA Kit-EHALB (Thermofisher, Waltham, MA, USA) according to the protocol provided by the manufacturer. The analyses were made on a Victor Nivo plate reader (Perkin Elmer, Waltham, MA, USA).

### 2.8. Assessment of the Ability of Cells to Synthesise and Store Glycogen

Some cultured cells were fixed with 4% PFA for 10 min at room temperature. The cells were stained for glycogen using the PAS method. In the first stage, the cells were treated with an oxidant 1% periodic acid for 5 min at room temperature, and then placed in Schiff’s reagent for 15 min at room temperature. Staining intensity was assessed under a light microscope. Cultured human dermal fibroblasts served as negative control.

### 2.9. Real-Time Quantitative Polymerase Chain Reaction (RT-qPCR)

The High Pure RNA Isolation Kit (Roche, Basel, Switzerland) was used to isolate RNA. The concentration and purity of the obtained RNA were measured with a NanoDrop ND-1000 spectrophotometer (Thermo Scientific, Waltham, MA, USA). The Transcriptor First Strand cDNA Synthesis Kit (Roche) was used for cDNA synthesis. The reaction was carried out in a T100 Thermal Cycler (Bio-Rad, Hercules, CA, USA). The RT-PCR reaction was performed using the LightCycler 480 Probes Master kit (Roche) and 50 ng of cDNA per reaction well. TaqMan probes (Thermofisher) coupled to the FAM dye were used in the reaction. The *PPIH* reference gene was selected by evaluating the expression of three potential reference genes—*GAPDH*, *RPLP0*, and *PPIH*—in eight randomly selected time points during cultivation, in both the control and the test groups. The TaqMan DNA Template Reagents Kit (Applied Biosystems, Waltham, MA, USA) was used to construct the standard curve. A real-time PCR reaction was performed to assess the expression of 13 genes (Table 2).

### 2.10. Statistical Analysis

The data were analysed using Statistica 13.0 software (StatSoft, Kraków, Poland). The results were presented as means and standard deviations (SD). In cases where the test results showed that the test sample met the conditions for normal distribution and homogeneity of variance, a two-factor ANOVA test was performed along with Tukey’s post hoc test. Student’s *t*-test for dependent and independent groups was also used where appropriate. The differences were considered statistically significant at *p* < 0.05. The results were presented graphically using GraphPad Prism 8.0 software (GraphPad, San Diego, CA, USA). LightCycler 96 SW 1.1 software (Roche) was used to analyse the RT-PCR data. RefFinder online tool [35] was used to assess the stability of the reference genes. This program assesses the stability of reference genes based on the algorithms of four other well-known programs: BestKeeper, NormFinder, Genorm, and the ΔΔCt comparison method.

## 3. Results

### 3.1. Cell Morphology

**Figure 1:**
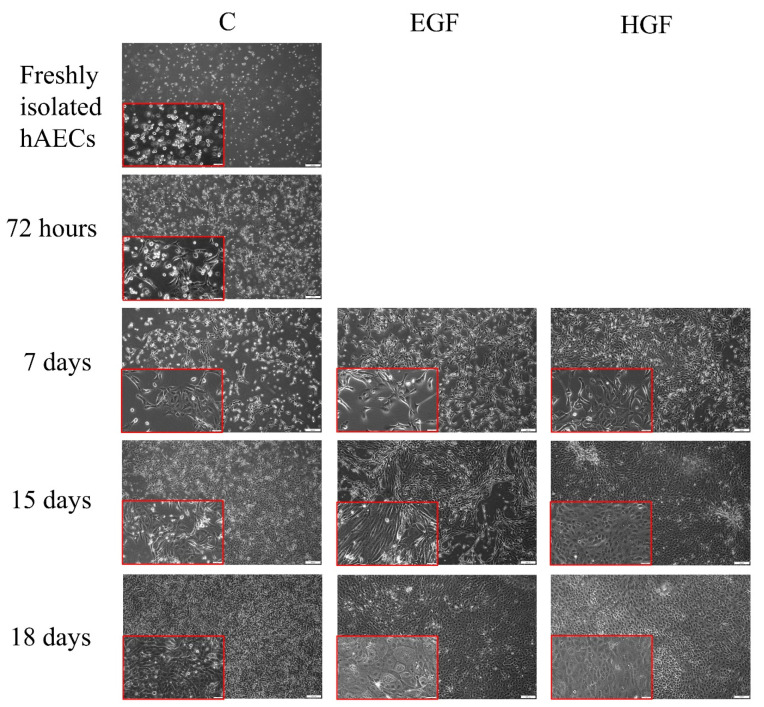
Comparison of the morphology of native hAECs in primary culture and in the control group as well as those differentiated in EGF- and HGF-supplemented media. Magnification—100×; scale—100 µm. In red frames: magnification—200×; scale—50 µm.

Trypsinization of the amniotic membrane resulted in a population of hAEC cells characterised by a small diameter, round shape, and round nucleus (Figure 1). After 72 h of pre-culture in DMEM/F12 medium, some of the seeded hAECs showed characteristics of adherent cells, which had an elongated spindle shape. On days 15 and 18 of culture, these cells had a large nucleus, sparse cytoplasm, and formed a cobblestone-like structure. In the differentiated groups, in both EGF- and HGF-supplemented media, single cells characterised by a polygonal shape, abundant cytoplasm, large nucleus, numerous dark granules in the cytoplasm, and the presence of large bright vacuoles resembling fat droplets was observed from day 7 and their number increased with culture time.

During cultivation, almost all cells expressed cytokeratin 7, a marker of epithelial cells. High expression of cytokeratin 7 was observed both in DMEM/F12 control medium and in differentiation media supplemented with HGF or EGF (See also Section 3.5).

### 3.2. Cell Count and Viability Assessment

**Figure 2:**
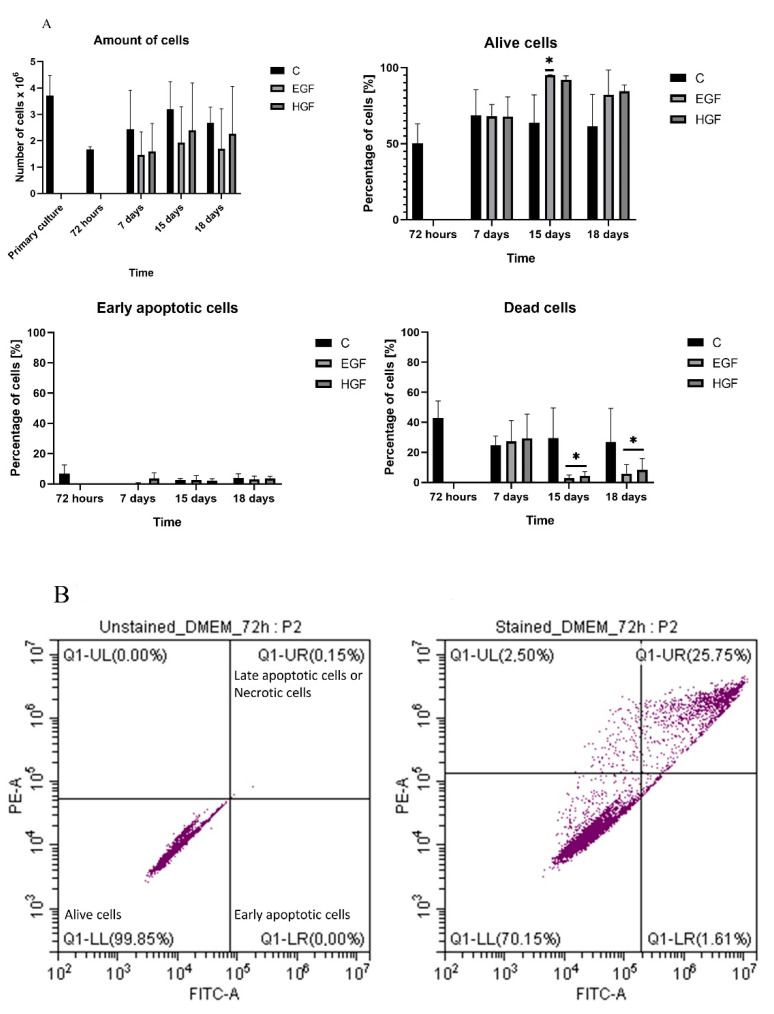
(**A**) The cell count and viability of hAECs in the control culture (C) and in the test groups (EGF and HGF) expressed as the percentage of viable cells—negative for annexin and propidium iodide; cells in early apoptosis—annexin-positive and propidium-iodide-negative cells; and dead cells—positive for both dyes. The mean number of seeded cells in each of the test groups was 3.7 million. * = statistically significant relative to control cells at 72 h (*p* < 0.05); *n* = 3. (**B**) The representative flow-cytometry histogram taken from control group at 72 h of culture shows the distribution of alive (LL), early apoptotic (LR), and late apoptotic/necrotic cells (UR). Left picture—unstained cells, right picture—stained cells.

In all groups, cell counts remained relatively constant between 72 h and 18 days of the control culture. The mean percentage of viable cells observed at 72 h (approx. 50%) showed an upward trend in the induced groups at subsequent time points. The percentage of cells in early apoptosis remained low in all culture groups (Figure 2).

### 3.3. Analysis of Expression and Co-Expression of Mesenchymal Surface Markers and the SSEA-4 Pluripotent Cell Marker

**Figure 3:**
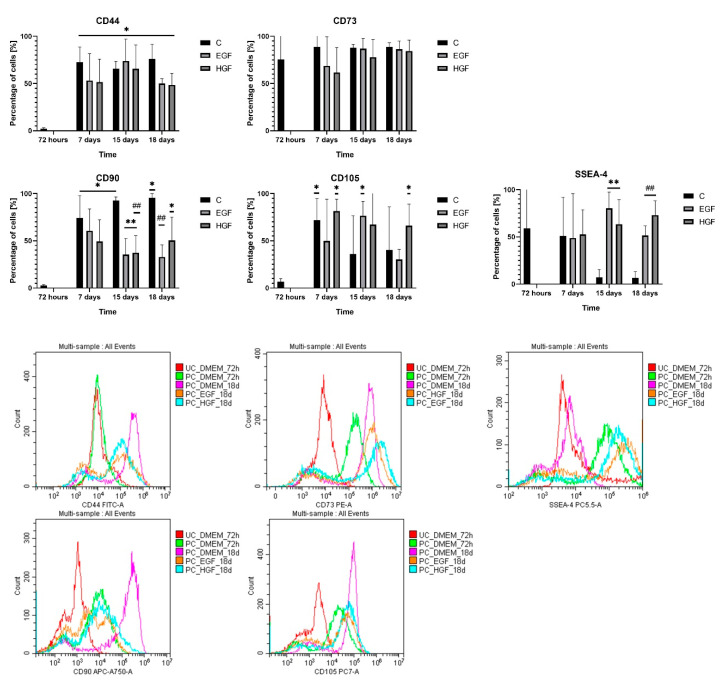
Percentage of CD44+, CD73+, CD90+, CD105+, and SSEA-4+ in the control culture (C) and subjected to differentiation (EGF and HGF). Statistically significant differences (*p* < 0.05) compared with group (C) at * = 72 h, ** = 15 days, and ## = 18 days; *n* = 3. The histograms present representative samples of fluorescence spectra in flow cytometry. UC—unstained cells; PC—stained cells.

At 72 h of culture, approx. 60% of native hAECs were SSEA-4+ cells, whose percentage in primary culture was subject to large individual fluctuations (Figure 3). In the control group, there was an approx. 80% decrease in the number of SSEA-4+ cells between days 7 and 15, while in both test groups, a high proportion of SSEA-4+ cells in the hAEC population was maintained throughout the duration of culture.

The primary hAEC population was characterised by low expression of CD44 (approx. 3% of the population), CD90 (<5%), and CD105 (6%). At subsequent time points, there was a significant increase in the percentage of CD44+ cells, 20-fold in the control group (*p* < 0.05) and 16–20-fold in the test groups compared with the native hAECs (*p* < 0.05). A similar upward trend was observed in the CD90+ and CD105+ counts (15–16-fold) in the control group between days 7 and 18 (*p* < 0.05). During this period, in the test groups, CD90+ counts were lower and CD105+ counts were comparable to the respective control groups. The percentage of CD73+ cells remained high (75%) in each group throughout the experiment.

Thus, the results of the performed determinations of mesenchymal cell surface markers and pluripotency marker during cultivation (Table 3) showed the following: (1) a decrease in the number of cells expressing SSEA-4 in the control group and its significant increase in both test groups; (2) a significant increase in the number of cells expressing CD44, CD90, and CD105 mesenchymal markers in the control culture; (3) a lower number of cells expressing mesenchymal markers in the test groups compared with the control culture, with the exception of CD105+ in the HGF group, which remained comparable at each time point.

As a result of such a distribution of the expression of the pluripotency marker and mesenchymal markers, at 72 h, the hAEC population was characterized by a small percentage (<2.3%) of cells with a phenotype combining pluripotency and mesenchymal cell characteristics (Table 4). During the control culture, the expression of cells with double-positive phenotypes increased, especially SSEA4+/CD73+ and SSEA4+/CD90+ cells, and at the same time SSEA-4−/CD44+ cells, which may indicate differential expression of mesenchymal markers on the cells of the disappearing SSEA4+ population and the emerging SSEA4− population. In contrast, the percentage of cells expressing SSEA-4 and simultaneously not expressing mesenchymal markers remained higher in the EGF- and HGF-induced groups compared with the respective control, except for the SSEA-4−/CD105.

### 3.4. Gene Expression Analysis of Pluripotency and Germ Layer Markers

**Figure 4:**
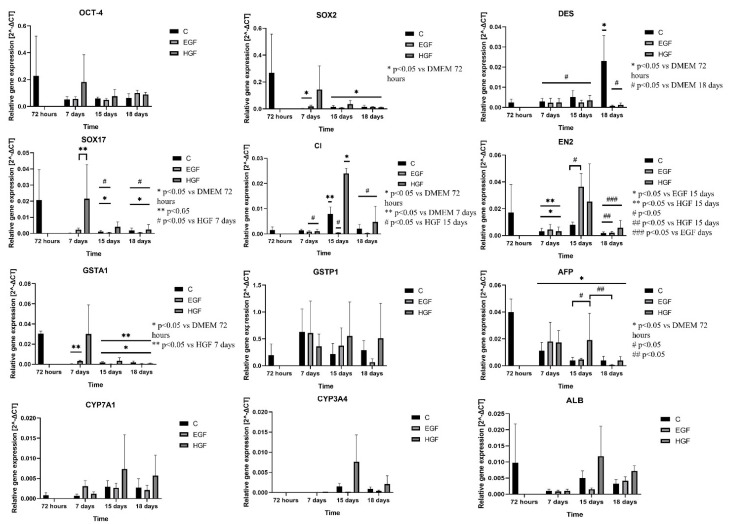
Expression of marker genes of pluripotency: *OCT-4* and *SOX2*; endoderm: *SOX17*; ectoderm: *EN2*; mesoderm: *DES*; haematopoietic cells: *CD45*. Genes of endoderm-derived cell lineages—*AFP*, *ALB*, *GSTA1*, *GSTP1*, *CYP3A4*, and *CYP7A1—*in the control group and in test groups assessed by the RT-PCR method. C—control; EGF and HGF—test groups; *n* = 3.

The *OCT-4* and *SOX2* genes responsible for pluripotency, *SOX17* gene representative of the endoderm, and *EN2* marker gene of endoderm differentiation showed the highest expression at 72 h. Expression of these genes decreased during cultivation in almost all groups and time points, especially for *SOX2* and *SOX17* (*p* < 0.05) (Figure 4). The highest expression of *EN2* was recorded in the induced groups on day 15 of culture. Expression of *DES* was initially low in native hAECs but increased by approx. 600% (*p* < 0.05) by day 18 of the culture. In the EGF- and HGF-induced groups at subsequent time points, *DES* expression remained at low levels, similar to or lower than in the control hAECs. In native hAECs, there was negligible expression of *CD45* except on day 15 of culture. At that time, in the HGF group, *CD45* expression was 15 times higher than the control level (*p* < 0.05).

In summary, the results of analysis of the expression of pluripotency genes and markers of differentiation into three germ layers showed the following (Table 5): (1) a decrease in pluripotency gene expression with culture time in the control group and expression stabilised at the control level in both test groups; (2) a significant, gradual increase in *DES* (mesodermal marker) expression in the control group with culture time and its gradual decrease in both test groups; (3) a decrease in *EN2* (ectodermal marker) expression in the control group and its increase in both test groups, especially on day 15 of culture; (4) a significant decrease in *SOX17* (endodermal marker) expression in the control culture and low level of its expression in both test groups on days 15 and 18; and (5) an increase in *CD45* (haematopoietic cell marker) expression in the control group and HGF, especially on day 15 of culture.

### 3.5. Expression Analysis of mRNA and Protein Markers of the Hepatoblast/Hepatocyte Lineage

At 72 h, native hAECs showed relatively high expression of genes characteristic of the hepatoblast lineage—*AFP* and *GSTA1*—but also of the *ALB* gene, a marker of the hepatocyte lineage. The expression of these genes decreased significantly with culture time. In almost all EGF- and HGF-treated cells, *AFP* expression was comparable to that of native hAECs. An upward trend in *ALB* gene expression was observed in the HGF group on days 15 and 18 of culture, but these changes were not statistically significant.

The *CYP3A4* hepatocyte lineage marker showed virtually no expression in native hAECs, while its expression was slightly increased on days 15 and 18 of culture in the HGF group. The expression of another marker, *CYP7A1*, remained at a comparable low level in the control and EGF groups until the end of the experiment and showed an upward trend in the HGF group on days 15 and 18 of culture. Changes in the expression of the *GSTP1* cholangiocyte marker in native hAECs were not statistically significant. There was a slight upward trend in expression levels in the HGF group on days 15 and 18 of culture.

**Figure 5:**
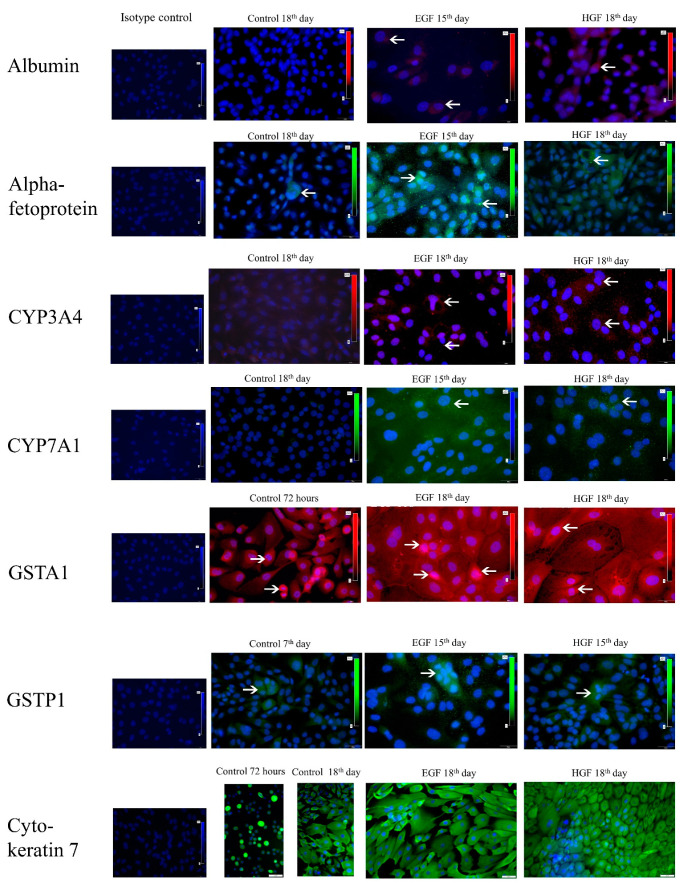
Immunofluorescent detection of hepatic markers—ALB, CYP4A4, CYP7A1; hepatoblast markers—AFP, GSTA1; cholangiocytic markers—GSTP1; and epithelial markers—CK7. Green colour—AFP, CYP7A1, GSTP1, CK7. Red colour—ALB, CYP3A4, GSTA1. The arrows indicate examples of positively stained cells. Appropriate isotype controls are also included. Magnification 400×.

**Figure 6:**
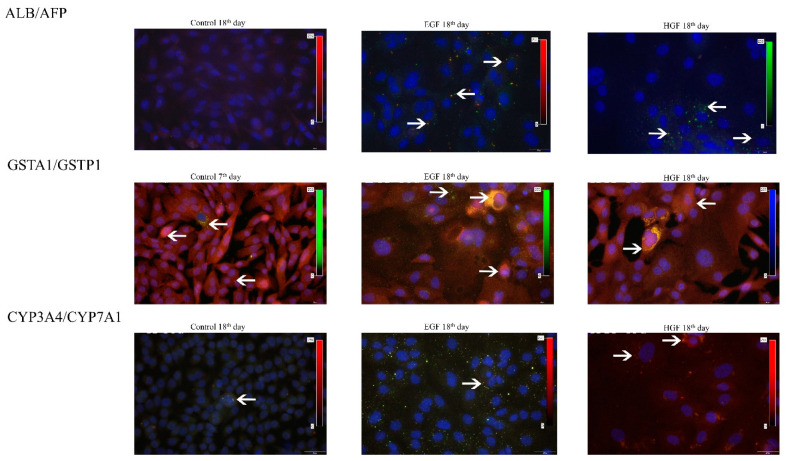
Co-expression of hepatic, hepatoblastic and cholangiocytic markers visualised immunohistochemically at 7 or 18 days of differentiation. Green colour—AFP, CYP7A1, GSTP1, CK7. Red colour—ALB, CYP3A4, GSTA1. Yellow/orange—double-positive cells. The arrows indicate examples of positively stained cells. Magnification 400×.

The results of the performed immunofluorescent staining of hepatic cell markers (Table 6, Figure 5) showed the following: (1) there was no albumin or alpha-fetoprotein in the primary hAEC population despite measurable levels of *ALB* and *AFP* gene expression. (2) Albumin was present in some cells of the test groups from day 15 of differentiation, with the highest number in the EGF group on day 18. (3) AFP+ cells were present in the HGF group from day 15 of culture and in the EGF group already from day 7 of culture. In the latter case, the percentage of AFP+ cells subsequently remained higher than in the HGF group. Most of the cells capable of synthesising albumin also synthesised significant amounts of alpha-fetoprotein (Figure 6). Both of these proteins localised in the cytoplasm, usually in close proximity to each other. (4) CYP3A4+ cells were present in each of the test groups, on days 15 and 18 under HGF supplementation, corresponding to an increase in *CYP3A4* gene expression in this group, and on day 18 in the EGF group. (5) The percentage of CYP7A1+ cells was higher in the EGF group than in the HGF group on days 7 and 15 and comparable on day 18. On day 15 of culture, both CYP3A4+/CYP7A− and double-positive CYP3A4+/CYP7A1+ cells were observed in the HGF group. On day 18 of culture, double-positive cells were observed in both test groups (Figure 6), while no cell with the CYP3A4−/CYP7A1+ phenotype were present in either test group. (6) There was a significant percentage of GSTA1+ cells in primary culture, decreasing in the control and test groups with culture time and a decrease in *GSTA1* gene expression. (7) The percentage of GSTP1+ cells was comparatively low in the HGF and control groups on days 7, 15, and 18, while it was high in the EGF group on day 7, gradually decreasing over the course of the experiment (Figure 6). Analysis of the co-expression of both types of glutathione S-transferase showed the presence of single cells with the GSTP1+/GSTA1− phenotype in the control group at 72 h and 7 days of culture, and also on day 7 of culture in the EGF group. No cells with this phenotype were observed in the other groups or time points. The majority of cells were double-negative cells, followed by cells synthesising only GSTA1 and single double-positive cells. GSTA1 localised centrally in the cytoplasm of cells as well as in the peripheral regions of the cytoplasm, in the vicinity of cell membranes. In contrast, GSTP1 tended to localise in the perinuclear region.

### 3.6. Functional Assessment of Differentiated Cells

**Figure 7:**
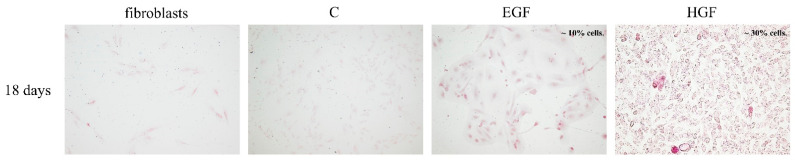
Detection of such glycogen presence in the test groups (PAS reaction). The colour reaction was positive in the EGF and HGF groups and negative in fibroblasts (negative control) and control hAECs (C). Magnification 100×.

The presence of glycogen was demonstrated in both test groups. In cells differentiated in HGF-supplemented medium, the percentage of PAS-positive cells was 20–30%, i.e., about three times more compared with the EGF group (Figure 7).

Human albumin was not detected in the culture medium in any of the test groups at the kit detection threshold of 6 ng/mL (not presented).

## 4. Discussion

In regenerative medicine, amniotic cells have become an attractive alternative to other stem cell populations due to the lack of moral and ethical controversies related to their procurement and the safety of their use in cell therapy. The unquestionable advantages of hAECs make them used in preclinical trials and clinical therapy of various diseases [25,36,37]. Unfortunately, they also have their limitations, such as their low capacity for division or loss of the stem cell phenotype in culture, making their large-scale use potentially difficult [38,39]. It is possible to increase ex vivo the proliferative and differentiating potential of hAECs by activation [40,41] and their partial pre-differentiation, which may be crucial for clinical use.

The process of hepatocyte differentiation is favoured by culturing in extracellular matrix-covered dishes. Substrates tested so far, including porcine-liver-derived extracellular matrix (L-ECM) [29], decellularized human liver extracellular matrix (hDLM) [42], laminins 411 (LN-411) and 521 (LN-521) [43], and the widely used Matrigel (Geltrex), i.e., the cell matrix derived from the Engelbreth-Holm-Swarm tumour [16,29,43,44,45], are characterised by varying effectiveness of differentiation. Some of them, e.g., LN-521, LN-411, and L-ECM, more efficiently increase the expression of genes encoding proteins from the cytochrome P450 family involved in the metabolism of endogenous compounds and xenobiotics [29,43]. Matrigel, on the other hand, is the most effective commercially available substrate, promoting the differentiation of stem cells into hepatocytes within 14 days, more than 90% of which are capable of albumin synthesis [15,45]. In contrast, hDLM, a highly effective differentiation medium, is not commercially available, and is acquisition requires a liver biopsy [42]. In addition, replacing foetal bovine serum (FBS), which is routinely used in cell culture, with KOSR, a serum-free cell medium, minimizes the participation of animal factors in the culture environment [46].

Protocols for differentiating stem cells into hepatocytes are continually being refined as the procedures so far have not had the expected full effect. Therefore, in our study, we attempted to use the proven protocol of hiPSC differentiation into hepatocytes [16] for the purposes of hAEC differentiation by comparing the effectiveness of high concentrations of epidermal and hepatocyte growth factors in stimulating the differentiation process. The original protocol was modified by eliminating the stage of cell differentiation into the definitive endoderm using iPSC-dedicated medium and by extending the pre-culture to 72 h. The changes made were aimed at increasing the viability of hAECs and obtaining from them quantitatively and quantitatively appropriate material.

After the first 72 h of pre-culture in control medium, hAECs on a Matrigel-coated substrate showed characteristics typical of culturing on this substrate [17,34,39,47,48]. A large proportion of the cells adhered to the culture dishes and some of them formed long cytoplasmic processes. Almost all cells expressed cytokeratin 7, a marker of epithelial cells [49]. To date, there is no consensus on the extent to which hAECs can express mesenchymal markers. It is known that they should be characterized by high expression of epithelial markers, e.g., cytokeratins [50], but at the same time they may show expression of mesenchymal surface markers, including almost 100% expression of CD73 [48,50], variable expression of CD90 ranging from 0% to approx. 30%, almost 60% expression of CD105 [39,48,50,51,52], and no expression of CD44 [48,53,54]. In the first 72 h of culture, we observed a high expression of CK7 and CD73, which together with low expression of CD44, CD90, and CD105 antigens and the *DES* gene confirmed the maintenance of the epithelial character of the isolated cells. At 72 h, the expression of the SSEA-4 surface marker and pluripotency genes (*OCT-4* (*POU5F1*) and *SOX2*) [55] was highest in native cells, confirming their initial stemness, then decreased with culture time. At the same time, only a small percentage of hAECs expressed *CD45*, possibly indicative of low contamination of the studied population with placental blood or vascular tissue [53]. In addition, hAECs were characterized by fairly high expression of endoderm (*SOX17*), hepatoblast (*AFP*, *GSTA1*), hepatocyte (*ALB*), and cholangiocyte (*GSTP1*) marker genes. It is difficult to say whether the expression of these genes is typical of native hAECs. Most studies indicate that these cells, when cultured in standard media, may express genes characteristic of the endoderm lineages or even be partially able to synthesize the proteins encoded by these genes [29,38,56,57,58]. It may be related to the region of the amniotic sac from which they were collected [59]. This phenomenon can be partially explained by the common origin of the placental tissues and the three germ layers from the epiblast [58]. However, some authors deny the possibility of expression of these genes and the corresponding proteins in amniotic epithelial cells [25]. We demonstrated that high expression of the *AFP* and *ALB* genes in native amniotic epithelial cells at 72 h of culture was not reflected at the proteome level. In contrast, high expression of the *GSTA1* gene was reflected in the expression of the GSTA1 enzyme involved in the detoxification processes. This enzyme is not a specific marker for foetal hepatocytes, since it is present both in hepatoblasts, in hepatocyte-like cells obtained in vitro by hESC differentiation [60], and in mature hepatocytes [61,62]. Furthermore, it is thought that analysis of changes in *GSTA1* gene expression may be useful in assessing the progression of EMT, during which it may increase [63]; however, we did not observe either at the mRNA or protein levels.

After 72 h of culture, the vast majority of control cells became elongated and spindle-shaped, similar to fibroblasts, which could indicate partial EMT [54,64]. From day 7, morphological changes were associated with increasing expression of the mesenchymal surface markers CD44, CD90, and CD105 and increasing expression of the *DES* gene. There were single cuboidal cells between the fibroblast-like cells, forming a cobblestone-like structure characteristic of epithelial cells [36,39], and CK7 expression remained high. Although a study by Pratama et al. from 2011 showed a decrease in CK7 expression with a simultaneous increase in the expression of mesenchymal markers, this occurred after a greater number of passages [54]. Between days 7 and 18 of culture, native hAECs showed stable viability and abundance and very high expression of CK7 and mesenchymal cell markers. These observations are analogous to the previous observations on long-term hAEC cultures [31,36,39,54].

CK7 expression remained high throughout hAEC differentiation, similar to the three-stage method using culture media supplemented with a mixture of EGF, FGF-2, and HGF [25]. At the same time, the expression of the desmin gene and mesenchymal surface markers increased slightly in the differentiated cells with culture time. This phenomenon relates to the common origin of both germ layers developing during gastrulation from the mesoendoderm and the co-occurrence of some markers [65] as well as the physiologically high expression of CK7 in both hepatocytes [66] and biliary epithelial cells [67]. In both induced groups, and especially in the HGF group, there was a significant increase in the expression of the two genes responsible for pluripotency (*SOX2* and *OCT-4*) and a persistently high percentage of SSEA-4+ cells. Previous research has shown that HGF stimulates an increase in *SOX2* and *OCT-4* gene expression in cancer stem cells via the c-MET receptor [68] and affects the maintenance of the stemness of human mesenchymal cells of bone marrow during long term cultures of over eight passages [69]. Based on our research it can be assumed that such a stimulating effect also occurs during short-term cultures.

According to the original hiPSC differentiation protocol, on day 7 of culture, the stem cells should differentiate into endodermal cells [16]. On day 7, in both induced groups (EGF and HGF), we observed single polygonal cells showing the presence of small dark cytoplasmic granules, which may correspond morphologically to definitive endoderm cells or, partially, to foetal hepatocytes [70,71]. After one week, HGF-treated hAECs showed a very significant increase in the expression of the transcription factor *SOX17*, as did hiPSCs treated with a dedicated medium differentiating into the definitive endoderm [16]. Taking this into account, and to increase hAEC viability without losing differentiation effectiveness, we omitted the stage of hAEC differentiation into definitive endoderm cells using the medium originally dedicated to iPSCs. Moreover, as early as day 7 of differentiation, a great number of cells expressed the AFP protein, which is characteristic of foetal hepatocytes, as well as CYP7A1, which is involved in cholesterol metabolism and is considered one of the markers of mature hepatocytes [72,73]. Previous studies have also found increased expression of the *CYP7A1* gene in hepatocyte-like cells resulting from successful differentiation of amniotic epithelial cells [29,34,74]. A similar effect of hAEC differentiation was achieved using a differentiation medium containing a 10-fold lower concentration of EGF and FBS;, however, this meant that it was not free of animal-derived factors [38]. Maymo et al. showed that this medium was extremely effective in promoting the differentiation of hAECs into hepatocytes, and already on the first day of differentiation, the resulting cells were able to synthesize albumin, alpha-fetoprotein, CYP3A4, and CYP7A1. However, it should be noted that the control hAECs also had some ability to synthesise these proteins at the same time. There was also a small but stimulating effect of low EGF concentrations (below 25 ng/mL) on the perinuclear localisation of HNF4A and thus a small effect on cell differentiation into hepatocytes. This is probably why supplementation of EGF control medium at a concentration of 20 ng/mL used in our model may have contributed to an increase in the expression of *CYP3A4* and *CYP7A1* genes but had no significant effect on the expression of proteins characteristic of hepatoblasts and hepatocytes, creating a significant difference between the control group and the group stimulated with a high dose of EGF. In our control culture, we found no presence of ALB, CYP3A4, or CYP7A1 proteins characteristic of the hepatocyte lineage. In contrast, HGF-treated cells showed less ability to synthesise alpha-fetoprotein as compared with the cells differentiated under the conditions of supplementation with EGF and hiPSCs treated with STEMDiff Definitive Endoderm [16].

Between days 7 and 18 of culture, in the groups supplemented with growth factors, we observed elongated spindle-shaped cells interspersed with large polygonal cells morphologically similar to hepatocytes obtained in hAEC differentiation by adapting the hiPSC differentiation protocol [13,33], based on a four-stage culture in differentiation media supplemented with activin-A, BMP4, FGF-2, HGF, oncostatin M, and sodium taurocholate hydrate. According to the original hiPSC differentiation protocol, on day 15 of culture, cells would be obtained with the characteristics of foetal hepatocytes, i.e., cells capable of synthesising AFP and HNF4A proteins but unable to synthesise albumin or cytochromes P450 and lacking typical hepatocyte morphology [16]. Our modifications allowed us to obtain, on day 15, in both test groups, approx. 15% cells showing typical hepatocyte morphology, capable of producing albumin, alpha-fetoprotein, CYP7A1, and CYP3A4 in the HGF group. This indicates that the applied modifications accelerated the process of differentiation of some cells.

We found no differences in hAEC morphology between EGF- and HGF-induced cells further differentiated after the foetal hepatocyte stage, i.e., after day 15 of culture. In both groups, extensive areas of culture dishes were covered with large polygonal cells with abundant cytoplasm-containing dark granules localised mainly perinuclearly, indirectly indicating the ability of the obtained cells to synthesise glycogen, which was confirmed on day 18 of culture by histochemical staining [75]. In some hepatocyte-like cells, there were large bright vacuoles possibly corresponding to fat droplets found in the cytoplasm of mature hepatocytes [76]. Similar results were achieved in other studies using the culture medium containing EGF, HGF, and FGF-4 in concentrations of 10 ng/mL as well as a 10% addition of conditioned medium from the culture of the HepG2 cell line [74].

At the end of the 18-day differentiation culture, the stemness potential of hAECs was not fully exhausted, as indicated by the high expression of SSEA-4 in differentiated cells. Differentiated amniotic epithelial cells showed higher viability than in the control culture and higher expression of the pluripotency marker SSEA-4, which is consistent with the results of Maymo et al. [38]. The expression of genes specific for hepatoblast (*AFP*) and hepatocyte (*CYP3A4*, *CYP7A1*, and *ALB*) lineages increased in both test groups, with the simultaneous appearance of proteins encoded by these genes in cells and decreasing (EGF group) or stabilised at a low level (HGF group) number of cells expressing the GSTP1 protein, one of the markers of biliary epithelial cells [77,78]. EGF-treated cells had the highest expression of alpha-fetoprotein on day 15, i.e., the assumed time of obtaining hepatoblasts, and the highest expression of albumin protein on day 18, when we expected to obtain hepatocytes. In contrast, in both test groups, we did not confirm the ability of cells to secrete albumin [33], but the analyses presented by Liu et al. provide an extremely extensive presentation of the functionality of the obtained cells through the use of in vivo tests, tumorigenicity tests, albumin and urea secretion, the ability to absorb ICG, evaluation of hepatic biochemical parameters, histopathological examination, and whole-body fluorescence imaging [25]. These studies clearly showed that hAECs even partially differentiated towards hepatocytes constitute an attractive therapeutic tool for treating liver failure.

In summary, isolated amniotic epithelial cells show expression of marker genes of the endoderm (*SOX17*) and the hepatoblast/hepatocyte lineage (*AFP*, *ALB*, *GSTA1*), which disappears during 18 days of native cell culture; they show trace or no expression of cytochrome P450 marker genes of mature hepatocytes; finally, they show stabilised expression of the *GSTP1* marker gene of cholangiocytes; however, only GSTA1 and GSTP1 expressions were measurable at the protein level. During 18 days of hAEC culture, high doses of EGF and HGF show a partially inhibitory effect on EMT between days 7 and 18 of hAEC culture. By modifying the differentiation protocol for hAEC culture, we obtained hepatocyte-like cells maintaining high expression of the marker of epithelial cells CK7; fairly high expression of mesenchymal markers CD44, CD90, CD73, and CD105; and partially expressing ALB, AFP, and cytochromes P450. As a result, the obtained cells showed an intermediate phenotype combining the characteristics of hepatoblasts and hepatocytes. Moreover, the high expression of mesenchymal surface markers CD44, CD73, CD90, and CD105, as well as the expression of *ALB* and *AFP*, may suggest that the obtained cells are similar to human liver stem-like cells [27,79,80,81], while increased expression of mesenchymal markers and cytochromes P450 brings them phenotypically close to adult-derived human liver mesenchymal-like cells [82]. The performed determinations showed that HGF was more effective than EGF in increasing the expression of genes characteristic for the hepatoblast/hepatocyte lineage in hAECs, which after 18 days of differentiation allows for the efficiency of hAEC differentiation into hepatocytes measured by the percentage of cells capable of synthesis albumin of approx. 20% and of synthesising glycogen at a level of approx. 30%. In contrast, EGF was more effective than HGF in influencing the expression of proteins characteristic of the hepatoblast/hepatocyte linage in hAECs, which allows for a high differentiation efficiency measured by the percentage of cells expressing protein markers of the hepatoblast lineage of approx. 50% already after 7 days of differentiation and by the percentage of cells capable of synthesising albumin at a level of approx. 40% and glycogen at a level of approx. 10% after 18 days of differentiation.

Thus, under differentiation conditions similar to those of induced pluripotent cells, it is possible to partially differentiate hAECs into hepatocytes. However, given the number of cells capable of synthesising albumin, the efficiency of hAEC differentiation into hepatocytes—using the modified hiPSC differentiation protocol [16]—was lower compared with the original method.

## Figures and Tables

**Table 1:** The two-stage hAECs differentiation protocol used to differentiate hAECs into liver cells. The cell analysis included cell count and viability using the ApoFlowEx FITC Kit (FACS); percentage of cells expressing the pluripotency marker and mesenchymal markers (FACS); percentage of cells capable of producing epithelial cell-specific proteins, hepatoblasts, hepatocytes, and cholangiocytes (ICC); expression of 13 genes characterising the degree of hAEC differentiation; and ability to synthesize and secrete albumin into the culture medium (ELISA) and to synthesise and store glycogen (PAS staining).

Experiment Duration										
0 h		72 h	3–6 Day	7 Day	8–10 Day	11 Day	12–14 Day	15 Day	16–17 Day	18 Day
**Cell seeding and culture in DMEM + ROCK; coating matrigel**										
		**Medium change and dividing cells into 2 test groups and 1 control group**						**Medium change**		
		**Control group:** DMEM/F12 + KOSR + NEAA + glutamine + pen/strep + 20 ng/mL EGF, coating: matrigel								
		**HGF group:** DMEM/F12 + KOSR + NEAA + glutamine + pen/strep + DMSO + **100 ng/mL HGF**, coating: matrigel						**HGF group:** DMEM/F12 + KOSR + NEAA + glutamine + pen/strep + dex, coating: Matrigel		
		**EGF group:** DMEM/F12 + KOSR + NEAA + glutamine + pen/strep + DMSO + **100 ng/mL EGF**, coating: matrigel						**EGF group:** DMEM/F12 + KOSR + NEAA + glutamine + pen/strep + dex, coating: Matrigel		
		**Naive hAEC analysis**		**Cell analysis**				**Cell analysis**		**Cell analysis**

**Table 2:** List of tested genes.

Gene	Full Gene Name	Group	Id Number (Thermofisher)
GAPDH	glyceraldehyde 3-phosphate dehydrogenase	Reference genes	Hs02786624_g1
POLR2A	RNA polymerase II subunit A		Hs00172187_m1
PPIA	peptidylptolyl isomerase H		Hs04194521_s1
POU5F1 (OCT-4)	octamer-binding transcriptor factor 4/POU class 5	Pluripotency related transcription factors	Hs04260367_gH
SOX2	sex determining region Y-box 2		Hs01053049_s1
PTPRC (CD45)	protein tyrosine phosphatase receptor type C	Hematopoietic stem cells marker	Hs04189704_m1
DES	desmin	Mesenchymal cells marker	Hs00157258_m1
EN2	engrailed homeobox 2	Ectodermal cells marker	Hs00171321_m1
HNF4A	hepatocyte nuclear factor 4 alpha	Endodermal cells markers	Hs00230853_m1
SOX17	SRY-Box transcription factor 17		Hs00751752_s1
AFP	alpha-fetoprotein	Hepatoblasts markers	Hs01040598_m1
GSTA1	glutathione S-transferase alpha 1		Hs07292901_gH
ALB	albumin	Hepatocytes markers	Hs00609411_m1
CYP7A1	cytochrome P450 family 7 subfamily A member 1		Hs00167982_m1
CYP3A4	cytochrome P450 family 3 subfamily A member 4		Hs00604506_m1
GSTP1	glutathione S-transferase Pi 1	Cholangiocyte marker	Hs00943350_g1

**Table 3:** Percentage change in the number of cells expressing pluripotency and mesenchymal surface markers in the control culture (compared with 72 h) and in differentiating cultures (EGF, HGF) relative to the respective control cultures.

	Control			EGF			HGF		
	7th Day	15th Day	18th Day	7th Day	15th Day	18th Day	7th Day	15th Day	18th Day
SSEA-4	**∨ 13.27%**	**∨ 87.8%**	**∨ 88.9%**	**∨ 4.43%**	**∧ 1017.4%**	**∧ 688.38%**	**∧ 3.13%**	**∧ 782.87%**	**∧ 1017.58%**
CD44	**∧ 3460.29%**	**∧ 3112.74%**	**∧ 3623.52%**	∨ 26.66%	∧ 12.74%	∨ 34.12%	∨ 29%	∧ 0.2%	∨ 36.08%
CD73	∧ 17.65%	∧ 16.18%	∧ 17.29%	∨ 22.70%	∨ 1.06%	∨ 13.64%	∨ 30.64%	∨ 11.32%	∨ 15.53%
CD90	**∧ 2547.5%**	**∧ 3205.71%**	**∧ 3311.42%**	∨ 18.22%	**∨ 52.42%**	**∨ 55.77%**	∨ 33.32%	**∨ 59.75%**	∨ 47.15%
CD105	**∧ 973.2%**	∧ 435.48%	∧ 504.8%	∨ 30.46%	∧ 114.2%	∨ 25.44%	∧ 13.61%	∧ 88.15%	∧ 63.73%

∧ = increase and ∨ = decrease in the number of positive cells; statistically significant changes are marked in bold (*p* < 0.05).

**Table 4:** Percentage of hAEC cells co-expressing SSEA-4 with mesenchymal markers CD44, CD73, CD90, and CD105 in the control group and differentiated groups (EGF and HGF). Statistically significant differences (*p* < 0.05) in relation to group (^a^) control 72 h, (^b^) control 7 days, (^c^) control 15 days, (^d^) control 18 days, (^e^) EGF 7 days, (^f^) EGF 15 days, (^g^) EGF 18 days, (^h^) HGF 7 days, (^i^) HGF 15 days, and (^j^) HGF 18 days; *n* = 3.

Phenotype		Control				EGF			HGF		
		72 h	7th Day	15th Day	18th Day	7th Day	15th Day	18th Day	7th Day	15th Day	18th Day
SSEA-4+	CD44+	2.18%	19.75%	**0.01%** ^f^	**1.12%** ^f^	45.36%	**71.39%** ^a^	31.18%	40.11%	50.62%	27.7%
	CD44−	10.52%	**4.16%** ^i^	**0.01%** ^i^	**0.02%** ^i^	**6.4%** ^i^	**11.12%** ^i^	14.84%	**13.75%** ^i^	15%	**39.93%** ^a^
	CD73+	0.01%	58.68%	47.56%	48.51%	43.73%	**64.38%** ^a^	**58.24%** ^a^	39.7%	**48.76%** ^a^	**47.45%** ^a^
	CD73−	0.14%	**2.15%** ^a^	0.03%	0.06%	**0.24%** ^b^	**2.1%** ^ac^	**2.14%** ^ace^	**4.31%** ^abe^	**3.59%** ^ace^	**4.44%** ^abce^
	CD90+	0.1%	44.65%	45.86%	52.63%	41.93%	**31.33%** ^a^	**25.93%** ^a^	38.83%	20.42%	**38.74%** ^a^
	CD90−	0.44%	**10.71%** ^a^	**1.22%** ^b^	**1.11%** ^b^	3.84%	**41.37%** ^abcd^	**39.13%** ^abcd^	**8.51%** ^a^	**30.17%** ^abcd^	**17.77%** ^acd^
	CD105+	2.3%	14.66%	**0.02%** ^a^	**0.01%** ^a^	32.69%	67.49%	21.88%	33.17%	54.47%	61.14%
	CD105−	**2.73% ^g^**	**12.23%** ^a**g**^	**0.2% ^g^**	**1.39% ^g^**	**2.67%** ^g^	**1.36%** ^**g**^	1.22%	**13.58%** ^**g**^	**1.98% ^g^**	**2.55% ^g^**
SSEA-4−	CD44+	1.08%	**47.46%** ^a^	59.93%	**78.03%** ^a^	**10.29%** ^dj^	**2.23%** ^bd^	**24.38%** ^d^	**26.46%** ^dj^	**5.55%** ^d^	**1.9%** ^bdj^
	CD44−	67.51%	23.98%	39.81%	18.45%	38.41%	13.11%	32.53%	34.89%	24.52%	28%
	CD73+	46.36%	17.91%	43.42%	53%	21.82%	20.72%	21.42%	**14.95%** ^a^	22.53%	24.66%
	CD73−	53.26%	16.4%	**7.6%** ^a^	**5.7%** ^a^	31.1%	12.97%	16.75%	31.87%	18.24%	23.13%
	CD90+	73.81%	**10.89% ^a^**	45.93%	47.44%	22.23%	**4.61%** ^a^	**7.02%** ^a^	**15.83%** ^a^	**10.23%** ^a^	**11.3%** ^a^
	CD90−	20.91%	24.18%	3.85%	4.18%	28.26%	20.94%	25.86%	32.04%	38.05%	31.58%
	CD105+	0.2%	1.78%	**0.2%** ^a**h**^	**0.01% ^h^**	0.8%	**6.26% ^h^**	**15.28%** ^**h**^	5.71%	5.94%	5.93%
	CD105−	94.11%	68.5%	99.82%	98.75%	56.03%	**23.15%** ^acd^	60.43%	43.8%	**34.87%** ^cd^	**28.96%** ^acd^

**Table 5:** The percentage change in the expression of marker genes of pluripotent cells (*OCT-4*, *SOX2*), mesenchymal cells (*DES*), ectodermal cells (*EN2*), endodermal cells (*SOX17*), and haematopoietic stem cells (*CD45*), in the control culture (compared with 72 h) and in differentiating cultures (EGF, HGF) relative to the respective control cultures.

	Control			EGF			HGF		
	7th Day	15th Day	18th Day	7th Day	15th Day	18th Day	7th Day	15th Day	18th Day
*OCT-4*	∨ 77.53%	∨ 74.45%	∨ 71.8%	∧ 9.8%	∨ 15.51%	∧ 56.25%	∧ 256.86%	∧ 29.31%	∧ 39.06%
*SOX2*	**∨ 99.02%**	**∨ 94.75%**	**∨ 94.38%**	∧ 592.3%	∨ 37.86%	∨ 0.04%	∧ 5438.46%	∧ 142.86%	∨ 27.47%
*DES*	∧ 20.38%	∧ 116.67%	**∧ 858.33%**	∨ 20.69%	∨ 53.84%	**∨ 96.52%**	∨ 17.24%	∨ 32.69%	**∨ 94.78%**
*EN2*	∨ 81.76%	∨ 54.11%	∨ 89.41%	∧ 41.93%	**∧ 361.53%**	∧ 5.55%	∧ 0.92%	∧ 223.07%	∧ 216.66%
*SOX17*	∨ 99.99%	**∨ 94.5%**	**∨ 91.5%**	∧ 2200%	∨ 58.62%	∨ 86.11%	∧ 21400%	∧ 253.44%	∧ 33.33%
*CD45*	∨ 7.33%	∧ 420%	∧ 33.33%	∨ 36.43%	∨ 94.29%	∨ 81.5%	∨ 28.57%	∧ 202.91%	∧ 135%

∧ = increase and ∨ = decrease in expression; statistically significant changes are marked in bold (*p* < 0.05).

**Table 6:** Phenotypic characterisation of hAECs in control culture (C) and differentiating cultures (EGF, HGF) based on the percentage change in gene expression and the number of cells expressing the relevant proteins—markers of hepatoblasts, hepatocytes, and cholangiocytes.

	Control				EGF			HGF		
Time Point Protein/Gene	72 h	7th Day	15th Day	18th Day	7th Day	15th Day	18th Day	7th Day	15th Day	18th Day
*ALB*	U	∨ 89.69%	∨ 48.45%	∨ 67.01%	∨ 18%	∨ 70%	∧ 28.08%	∧ 3.96%	∧ 136%	∧ 122.22%
Albumin	-	-	-	-	-	+	++	-	+	+
*AFP*	U	**∨ 72.5%**	**∨ 90.5%**	**∨ 90%**	∧ 63.63%	∧ 21.05%	∨ 85%	∧ 54.54%	**∧ 400%**	∨ 2.5%
Alpha-fetoprotein	-	-	-	-	+++	++	++	-	+	+
*CYP3A4*	-	0.00%	∧ 1400%	∧ 770%	0.00%	∨ 86%	∨ 54.02%	0.00%	∧ 409.33%	∧ 141.37%
CYP3A4	X	-	-	-	-	-	+	-	+	+
*CYP7A1*	U	∨ 19.04%	∧ 245.23%	∧ 221.43%	∧ 334.78%	∨ 10.34%	∨ 25.92%	∧ 73.91%	∧ 151.72%	∧ 111.11%
CYP7A1	-	-	-	-	++	+	+	+	+	++
*GSTA1*	U	∨ 99.98%	**∨ 94.33%**	**∨ 93.66%**	∧ 3100%	∨ 99.97%	∨ 73.68%	∧ 29900%	∧ 100%	∨ 62.63%
GSTA1	++++	++	+	+	++	+	+	++	+	+
*GSTP1*	U	∧ 221.53%	∧ 10.25%	∧ 47.69%	∨ 3.35%	∧ 72.56%	∨ 77.43%	∨ 42.74%	∧ 156.74%	∧ 76.39%
GSTP1	-	+	+	+	+++	++	+	+	+	+
Cytokeratin 7	++++	++++	++++	++++	++++	++++	++++	++++	++++	++++

Italics—gene names; (X)—unmeasurable gene expression; (U)—measurable gene expression at first time point; ∧ = increase and ∨ = decrease in gene expression; (-)—no protein or single-positive cells < 1%; (+)—protein present in 1–25% cells; (++)—protein present in 26–50% cells; (+++)—protein present in 51–75% cells; (++++)—protein present in 76–100% cells; bold—statistically significant changes (*p* < 0.05) compared with 72 h (in the control group) and to the respective control groups (in the EGF and HGF groups).

## Data Availability

Not applicable.
